# Prioritizing Therapeutic Targets for Interstitial Lung Disease: A Causal Mediation Analysis

**DOI:** 10.21203/rs.3.rs-8714555/v1

**Published:** 2026-02-05

**Authors:** Justin Oldham, Philip Molyneaux, Manoj Maddali, Chad Newton, John Kim, Sam Konkol, Janelle Pugashetti, Gabrielle Liu, Gillian Goobie, Ayodeji Adegunsoye, Shwu-Fan Ma, Drew Bornstein, Susan Murray, Louise Wain, Gauri Saini, Iain Stewart, Simon Johnson, Gisli Jenkins, Mary Strek, Angela Linderholm, Ching-Hsien Chen, William Fahy, Rachel Zemans, Bethany Moore, Dinesh Khanna, Christopher Ryerson, Kevin Flaherty, Madathilparambil Suresh, Anna-Maria Hoffmann-Vold, Toby Maher, Christine Garcia, Paul Wolters, Fernando Martinez, Imre Noth, Jennifer A. Smith

**Affiliations:** University of Michigan; National Heart and Lung Institute, Imperial College London; Piedmont Healthcare; University of Texas Southwestern; University of Virginia; University of Virginia; University of Michigan; University of California at Davis; University of British Columbia; University of Chicago; University of Virginia; University of Michigan; University of Michigan; University of Leicester; University of Nottingham; Imperial College London; University of Nottingham; National Heart and Lung Institute, Imperial College; University of Chicago; University of California at Davis; University of California Davis; GlaxoSmithKline (United Kingdom); University of Michigan–Ann Arbor; University of Michigan; University of British Columbia; University of Michigan; University of Michigan–Ann Arbor; Oslo University Hospital; Royal Brompton Hospital; Columbia University Medical Center; University of California, San Francisco; University of Massachusetts Chan Medical School; University of Virginia School of Medicine; Department of Epidemiology, School of Public Health, University of Michigan, Ann Arbor, MI, USA

**Keywords:** Interstitial Lung Disease, Progressive Pulmonary Fibrosis, Proteomics, Biomarker, Mediation

## Abstract

Progressive interstitial lung disease (ILD) leads to declining lung function and death. New therapies to treat ILD are urgently needed. Here we performed a secondary analysis of proteomic data from ten ILD cohorts across the United States, Canada, and United Kingdom. Causal mediation analysis was used to estimate the effect of plasma proteins previously linked to organ fibrosis in mechanistic studies (exposure) on survival (outcome) through lung function decline (mediator). Of 102 proteins tested in a discovery cohort (n = 1963), 47 were mediated by declining lung function. Of these 47 proteins, 7 showed sustained mediation in an independent validation cohort (n = 1172). Proteins with the strongest mediated effect were amphiregulin and integrin beta six. Sensitivity analysis showed that results were robust to unmeasured confounding. Here we provide epidemiological evidence implicating seven proteins as potentially causal of progressive ILD. These findings build upon mechanistic studies showing a causal link between these proteins and organ fibrosis, supporting their prioritization for therapeutic consideration.

## Introduction

When progressive, interstitial lung disease (ILD) typically leads to declining lung function and death.^1^ With a median survival of less than 5 years following diagnosis, idiopathic pulmonary fibrosis (IPF) is considered the most progressive ILD subtype.^2^ However, large proportions of non-IPF ILDs also progress, including connective tissue disease-associated ILD, unclassifiable ILD, fibrotic hypersensitivity pneumonitis and idiopathic non-specific interstitial pneumonia, leading to similarly poor survival.^2–4^ Nintedanib and, more recently, nerandomilast are approved for the treatment of progressive ILD after pivotal trials demonstrated efficacy in slowing lung function decline.^5–8^ While these drugs, along with pirfenidone,^9^ represent an important advance for the field, none stop or reverse lung fibrosis, highlighting the urgent need for new therapies.

**Figure 1:**
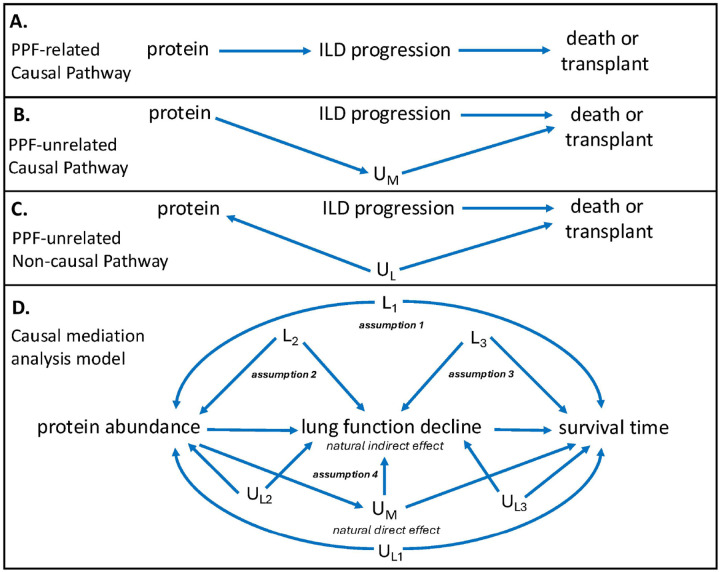
Causal framework depicting an association between a protein and ILD survival through an ILD-related causal pathway (a), ILD-unrelated causal pathway (b), and non-causal pathway due to residual confounding (c), along with causal diagram depicting modeling approach to satisfy key assumptions of mediation analysis. Abbreviation: ILD = interstitial lung disease; L_1_ = measured confounders of exposure-outcome relationship, L_2_ = measured confounders of exposure-mediator relationship, L_3_ = measured confounders of mediator-outcome relationship, U_M_ = unmeasured mediator; U_L1–3_ = unmeasured confounders of exposure-outcome, exposure-mediator and mediator-outcome relationship. Mediator model covariates selected were age, sex, race, smoking history, body mass index, ILD diagnosis, baseline FVC, baseline DLCO and anti-fibrotic and immunosuppressant exposure at the time of blood draw. Outcome model covariates selected were mediator model covariates plus anti-fibrotic and immunosuppressant exposure following blood draw.

New drug development for ILD has proven challenging, as few drivers of organ fibrosis identified through mechanistic studies have translated into effective human therapies. Human-based association studies can help increase confidence in mechanistic findings, but themselves cannot establish causation. This limitation hampers translation because protein-outcome associations can arise through a disease-related causal pathway (Fig. 1A), a disease-unrelated causal pathway (Fig. 1B) and a non-causal pathway related to residual confounding (Fig. 1C). Causal mediation analysis is an epidemiological method designed to overcome this uncertainty. This approach deconstructs the direct and indirect effects of an exposure on an outcome, with the indirect effects occurring through a third variable called a mediator.^10^

We recently used causal mediation analysis to show that chronological age has no direct effect on ILD survival, which is instead mediated by biological age, as measured by aging biomarkers.^11^ A similar approach could potentially clarify the pathways in which a circulating protein biomarker associates with ILD survival. Here we leveraged proteomic and phenotypic data from ten prospective ILD registries and cohort studies to conduct causal mediation analysis aimed at identifying circulating proteins potentially causal of ILD progression in humans, thereby prioritizing these proteins for therapeutic consideration. We hypothesized that causal mediation analysis would discriminate proteins that associate with ILD survival through declining lung function (Fig. 1A), a cardinal feature of progressive ILD,^3,12^ from those that associate with this outcome through one or more alternate pathways (Figs. 1B–C). Proteins mediated by lung function decline across two independent, multicenter ILD cohorts and causal of organ fibrosis in laboratory models were classified as candidate therapeutic targets.

## Results

### Cohort characteristics

Of 2693 and 1324 eligible individuals who underwent proteomic profiling in the discovery and validation cohorts, respectively, 1963 (73%) and 1172 (89%) were included in the analysis (**Figure E1**). The mean age was 66 years in both cohorts, and a majority of individuals were male, white and reported a history of smoking cigarettes (Table 1). IPF was the predominant diagnosis in the discovery cohort, while similar proportions of IPF and CTD-ILD comprised the validation cohort. Mean percent predicted FVC and DLCO were higher and a larger proportion was treated with anti-fibrotic therapy in the discovery cohort, while a larger proportion was treated with immunosuppressant therapy in the validation cohort.

### Outcomes, Lung Function Decline and Composite Measure of Lung Function Decline

**Figure 2:**
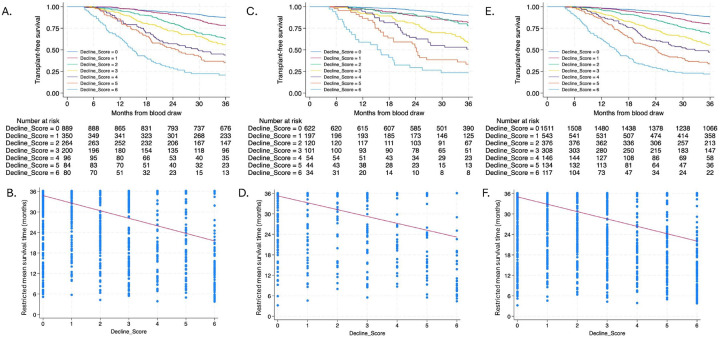
Relationship between lung function decline score and three-year transplant-free survival and restricted mean survival time in the discovery (A-B), validation (C-D) and pooled (E-F) cohorts.

During the 36-month observation period, 520/1963 (26.5%) and 237/1172 (20.2%) individuals died or underwent lung transplant in the discovery and validation cohorts, respectively. Mean annualized FVC decline was 8.4% (±13.8%) in the discovery cohort and 7.4% (±12.7%) in the validation cohort, while mean annualized DLCO decline was 14.9% (±20.6%) and 11.5% (±17.8%), respectively. The composite measure of lung function decline ranged from a score of 0–7, with increasing score negatively correlated with transplant-free survival and RMST (Fig. 2). Each unit increase in lung function decline score was associated with a 1.96 (coefficient − 1.96; 95% CI −2.14, −1.78) and 1.70 (coefficient − 1.70; 95% CI −1.93, −1.48) month decrease in RMST in the discovery and validation cohorts, respectively.

### Causal Mediation Analysis

Of 185 proteins with previously published association with ILD survival, 102 had previously published mechanistic evidence suggesting a role in organ fibrosis (**Table E2**). In the discovery cohort, when conducting causal mediation analysis of these 102 proteins, 67 were associated with RMST at total effect FDR p < 0.05. Declining lung function mediated the RMST association at NIE FDR p < 0.05 for 47 of these 67 proteins, with the strongest mediated effect observed for amphiregulin (AREG) and integrin beta six (ITGB6) (Table 2, **Table E3**). When assessed in the validation cohort, 25/47 proteins showed sustained association with RMST at total effect FDR p < 0.05. Declining lung function mediated the RMST association at NIE FDR p < 0.05 for 7 of these 25 proteins, with AREG and ITGB6 again showing the strongest mediated effects (Table 2, **Table E4**). In addition to AREG and ITGB6, granulocyte-macrophage colony-stimulating factor (CSF2), growth differentiation factor 15 (GDF15), interleukin-5 receptor subunit alpha (IL5RA), stromelysin-2 (MMP10) and group 10 secretory phospholipase A2 (PLA2G10) had RMST association that was significantly mediated by declining lung function across discovery and validation cohorts. Thus, these proteins were considered potentially causal of ILD progression and identified as high-yield therapeutic targets.

**Figure 3:**
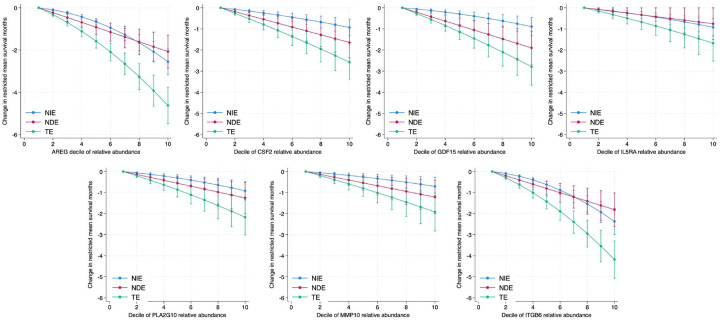
Effect plots depicting the total effect (TE), natural indirect effect (NIE) and natural direct effect (NDE) on restricted mean survival time (RMST).

After pooling discovery and validation cohorts, effect plots showed that mediated effects (NIE) increased with relative abundance of each validated protein (Fig. 3). Mediated effects predominated as AREG, ITGB6 and IL5RA relative abundance increased (Fig. 3). In subgroup analyses, mediated effects were similar for validated proteins when stratifying by age (**Table E6**) and race (**Table E7**), but higher among males (**Table E8**) and those with lower baseline percent predicted FVC (**Table E9**) and DLCO (**Table E10**). Heterogeneity was also observed across diagnostic subgroups. Mediated effects were less in those CTD-ILD compared to those with IPF and other forms of fibrotic ILD (**Table E11**).

In sensitivity analyses, results were similar when using alternative quantile normalization strategies, with higher mediated effects as quantile strata increased (**Table E12**). Results were also robust to DLCO imputation strategy, with significant mediation observed for all validated proteins after excluding those for which DLCO imputation was performed (**Table E13**). Confounding sensitivity analysis^13,14^ showed mediational E-values of approximately 1.5–2.0 for all validated proteins in each cohort (Table 2), suggesting that an unmeasured confounder of the mediator-outcome relationship with a risk ratio greater than this E-value would be required to attenuate results. Approximated risk ratios^13,14^ for known confounders of ILD outcome risk, including age ≥65 (aRR 1.02), male sex (aRR 1.03), IPF diagnosis (aRR 1.04), CTD diagnosis (aRR 0.94), baseline FVC < 70 (aRR 1.05) and baseline DLCO < 50% (aRR 1.06) were less than E-values for all validated proteins.

## Discussion

In this international multicohort study, we identified seven circulating plasma proteins whose associations with ILD survival are mediated through declining lung function, implicating these proteins as potential causal drivers of progressive pulmonary fibrosis. Subgroup analyses supported the biological plausibility of these findings, with stronger mediated effects observed in fibrotic-predominant ILDs (e.g., IPF and non-CTD ILDs) and among patients with more advanced disease, as measured by baseline lung function. Sensitivity analyses suggested that results were robust to quantile normalization strategy, imputation for missing DLCO and unmeasured confounding. By moving beyond traditional association analyses to interrogate causal pathways, this study provides human evidence linking these proteins to clinical outcomes through physiologic deterioration. Coupled with prior mechanistic work linking these proteins to organ fibrosis, this study supports prioritizing these proteins as promising therapeutic targets to treat progressive pulmonary fibrosis.

Among validated proteins, mediation was strongest for AREG across discovery and validation cohorts, along with most key subgroups. AREG is an epidermal growth factor receptor (EGFR) ligand that can activate transforming growth factor beta 1 (TGF-β1) and lead to fibrotic remodeling through EGFRmediated fibroblast activation.^15,16^ In the lungs, AREG has been implicated in macrophage-mediated tissue remodeling, with macrophages serving as a critical cellular source of AREG during tissue injury and repair.^17,18^ AREG blockade has been shown to attenuate pulmonary fibrosis in mouse models.^15,19^ A small interfering RNA targeting AREG is currently under development for fibrotic conditions after showing promising results in reducing kidney fibrosis,^20^ with a press release suggesting an acceptable safety profile from a recent phase I trial in healthy participants (NCT05984992). A monoclonal antibody targeting AREG is also under development, with recent phase 1b results suggesting an acceptable safety profile and beneficial effect on FVC and quantitative CT fibrosis in patients with IPF.^21^ Importantly, pneumonitis has not reported with AREG blockade, which remains a concern with direct EGFR inhibitors.^22^

ITGB6 was the second most strongly mediated protein in our analysis. ITGB6 makes up the β6 subunit of integrin αvβ6, which has long been causally linked to fibrogenesis. αvβ6 activates latent TGF-β1, leading to fibroblast-to-myofibroblast transition and collagen deposition in the lungs and elsewhere.^23^ Inhibition of αvβ6 has been shown to attenuate fibrosis in mice mouse models of fibrosis^24^ and slow IPF progression in an early phase clinical trial.^25^ However, recent phase II trials that targeted αvβ6 using monoclonal antibodies^26^ and a small molecule inhibitor (NCT06097260) were stopped due to safety concerns, suggesting that direct αvβ6 blockade may not be possible.

GDF15 is a secreted ligand of the TGF-β superfamily of proteins, which regulates energy expenditure and body weight in response to metabolic stress.^27–29^ This protein has been shown to increase with age and has been implicated in numerous aging-relating conditions, including cardiovascular disease, diabetes and chronic lung disease, including COPD and IPF.^30–33^ GDF15 is elevated in the lungs of patients with IPF where it likely facilitates extracellular matrix formation through direct fibroblast activation and differentiation.^34–36^

CSF2, is a granulocyte-macrophage colony stimulating factor that plays an important role in inflammation and tissue repair. CSF2 overexpression has been shown to stimulate TGF-β1 production by alveolar macrophages, which appears to be independent of inflammation-driven changes.^37^ Whether CSF2 blockade could attenuate fibrosis remains unclear however, as neutralizing anti-bodies worsened fibrosis severity in a mouse model of pulmonary fibrosis.^38^

PLA2G10 belongs to the family of secretory phospholipase A2 (PLA2) enzymes, which produce free fatty acids and lysophospholipids.^39^ While little is known about the role PLA2G10 may play in fibrogenesis, recent studies have shown that PLA2G10 is highly expressed in IPF lungs^40^ and different types of cancer.^41^ PLA2G10 upregulation also prevented T cell infiltration of cancer tissue, suggesting that PLA2G10 could represent a therapeutic target for cancer immunotherapy.^41^ Lysophosphatidic acid (LPA) is a well-recognized pro-fibrotic mediator and can be produced by autotaxin and PLA2.^42,43^ Autotaxin inhibition failed to slow IPF in a recent phase III clinical trial^44^ while LPA blockade is currently being investigated in phase III clinical trials for IPF and progressive non-IPF ILD after promising phase II data.^45^

MMP10 is a member of the matrix metalloproteinase family of proteins, playing a key role in cell adhesion, migration and proliferation during wound healing.^46^ Lung expression of MMP10 is increased in patients with IPF and has been shown to localize to alveolar and bronchiolar epithelium, along with pulmonary macrophages.^47^ While mechanistic studies establishing a causal relationship between MMP10 and pulmonary fibrosis have not been performed, a mouse model of peritoneal fibrosis suggests that MMP10 blockade may have anti-fibrotic effects^48^ and a recent early phase clinical trial showed that 12-week change in circulating MMP10 after treatment with rentosertib, a small molecule TNIK inhibitor, inversely correlated with change in FVC over the same timeframe.^49^

IL5RA is widely studied and well-established regulator of eosinophil activation and survival.^50^ An important contributor of eosinophilic-mediated conditions such as asthma, IL5RA also appears to drive subepithelial fibrosis in this population^51^ and blockade of this molecular reduces expression of several key extracellular matrix proteins, including tenascin C and procollagen III.^52^ recent studies have also demonstrated IL5RA receptor expression in bronchial fibroblasts,^53^ suggesting a potential role in parenchymal fibrogenesis. Single cell sequencing data support this possibility, showing upregulated IL5RA expression in pulmonary fibrosis, which promotes fibrogenesis through the Jak2/STAT3 pathway.^54^ Importantly, benralizumab, an anti-IL5RA monoclonal antibody is already approved for the treatment of severe eosinophilic-mediated conditions such as asthma and eosinophilic granulomatous with polyangiitis. Our data suggest that repurposing of this safe and well tolerated drug^55^ could potentially provide benefit for ILD.

Our study has several limitations. First, our study design was also prone to selection bias, as only patients with serial FVC measures were included, which likely selected for individuals with less severe and progressive disease. Next, our exposure, mediator and outcome variables were each prone to measurement error. For exposure measurement error, proximity extension assays detect low abundance proteins with excellent specificity, but some degree of cross reactivity remains possible. For mediator measurement error, declining FVC and DLCO represent cardinal features of progressive ILD,^3,12^ but do not fully explain this phenomenon, which can also manifest as increasing extent of fibrosis on chest imaging and worsening respiratory symptoms without lung function decline.^12^ The incomplete mediation observed in this analysis underscores the difficulty of establishing an optimal measure that accurately captures a progressive phenotype. For outcome measurement error, some patients will die from a competing cause of death rather than ILD.^56^ Each of these sources of measurement error likely attenuated results rather than biasing results, as none were likely differential by one another. Finally, despite a rigorous attempt to satisfy key assumptions of causal mediation analysis, residual confounding remains possible. However, our confounding sensitivity analysis suggested that unmeasured confounders with larger effect size than known confounders would be required to attenuate results.

## Conclusion

Through causal mediation analysis, this study identified a small number of prognostic protein biomarkers that are likely to play a causal role in progressive ILD. This study provides novel insights into ILD pathobiology and helps to prioritize proteins and associated molecular pathways for therapeutic consideration. While not all candidate causal biomarkers identified here represent viable therapeutic targets, our study showcases the role causal mediation analysis can play in prioritizing molecular targets for therapeutic consideration.

## Methods

### Cohorts, Data Generation and Protein Selection

Individuals with IPF, connective tissue disease-associated ILD (CTD-ILD), fibrotic hypersensitivity pneumonitis, idiopathic non-specific interstitial pneumonia and unclassifiable ILD who underwent high-throughput proteomic profiling as part of two recently published proteomic investigations^57,58^ and a new international proteomic cohort study were eligible for inclusion (**Table E1**). Those without baseline forced vital capacity (FVC) and diffusion capacity for carbon monoxide (DLCO) (range − 6 to + 3 months relative to blood draw), at least one FVC measure following blood draw (range 3–24 months), and complete data for covariates included in mediation modeling (see below) were excluded.

Methods for proteomic data generation have been described previously.^57,58^ Briefly, the Explore 3072 and HT arrays (Olink, Uppsala, Sweden) were used to generate proteomic data in the discovery and validation cohorts, respectively. These arrays use proximity extension assays to estimate the relative abundance of circulating plasma proteins.^59^ Quantile normalization was performed to harmonize proteomic data generated across different batches, with each protein categorized according to decile of relative abundance. To increase confidence in biologically plausible results, the analysis was restricted to proteins previously linked to ILD survival in human-based studies and organ fibrosis in mechanistic studies.

### Causal Mediation Analysis

Based on the causal framework depicted in Fig. 1, there is no direct causal pathway from a circulating protein to death in those with ILD without an intermediate process. Instead, a protein likely influences this outcome by contributing to ILD progression (Fig. 1A) or an unmeasured condition (Fig. 1B). A non-causal association between protein and outcome could also exist due to unmeasured confounding (Fig. 1C). To discriminate these pathways, causal mediation analysis was performed using the *mediate* package in STATA (version 18, College Station, TX).

Exposure was defined as decile of relative protein abundance and modeled as a continuous variable. Mediator was defined as degree of lung function decline and modeled as a continuous variable. To capture the prognostic significance of declining FVC and DLCO,^3,12^ a composite measure of annualized relative decline for both was developed (**supplementary methods**). Because missing DLCO measures can result from the inability to perform the maneuver, which has prognostic significance,^60^ imputation was performed to estimate the expected rate of DLCO decline when missing for those in the discovery (6.3%; 123/1963) and validation (9.9%; 116/1172) cohorts (**supplementary methods**). Outcome was defined as three-year restricted mean transplant-free survival time (RMST), which converts time-to-event data to a continuous measure for generalized linear modeling.^61^ RMST was estimated using the *stpmean* package in STATA, with transplant-free survival defined as the time from blood draw to death, lung transplant or censoring at 36-months or sooner if lost-to-follow-up.

The mediation model framework is depicted in Fig. 1D. To derive causal interpretations, mediation analysis assumes that there exists no confounding of the 1) exposure-outcome relationship, 2) the exposure-mediator relationship, 3) the mediator-outcome relationship, and 4) the mediator-outcome relationship caused by the exposure.^10^ To satisfy assumption two, the mediator model was adjusted for center, proteomic batch, age, sex, race, ILD diagnosis, smoking history, baseline percent predicted FVC and DLCO, pulmonary hypertension risk and exposure to anti-fibrotic (nintedanib or pirfenidone) and immunosuppressant (mycophenolate mofetil, azathioprine, rituximab or cyclophosphamide) therapy at the time of blood draw. To satisfy assumptions one and three, the outcome model was adjusted for these covariates plus new anti-fibrotic and immunosuppressant exposure following blood draw. To address assumption four, we utilized relatively short windows between exposure, mediator and outcome,^10^ which reduced the likelihood of death due to a competing condition.^56^

When reporting results, the total effect represents the RMST difference in months between groups in the first and tenth deciles of protein relative abundance. The natural indirect effect (NIE), also referred to as the mediated effect, represents the difference in RMST between these groups due to declining lung function (Fig. 1A). The natural direct effect (NDE) represents the difference in RMST between these groups due to an unmeasured pathway (Figs. 1B–C). Exposure-mediator interaction was allowed in all analyses, and robust standard errors were used when estimating effect estimates.

Because mediation analysis requires an exposure-outcome association and a plausible biological relationship between exposure, mediator and outcome, only proteins with total effect p < 0.05 after false discovery rate (FDR) adjustment using the Benjamini Hochberg procedure^62^ and previously linked to organ fibrosis in mechanistic studies were considered. Proteins associated with RMST through the lung function decline pathway (NIE FDR p < 0.05) in the discovery cohort were advanced for validation cohort testing. Those with sustained mediation by declining lung function in the validation cohort at NIE FDR p < 0.05 were considered potentially causal of progressive ILD and classified as candidate therapeutic targets. Discovery and validation cohorts were then pooled and effect plots generated to visualize mediated effects over the full range of protein values. Subgroup analyses were performed after stratification by key demographic, physiological, and diagnostic subgroups. Sensitivity analyses were performed to evaluate the effect of different quantile normalization strategies and exclusion of those with imputed DLCO decline values. Confounding sensitivity analysis was performed to estimate the mediational E-value for each protein, which estimates amount of residual confounding that would be required to attenuate results.^13,14^

## Supplementary Material

Supplementary Files

This is a list of supplementary files associated with this preprint. Click to download.

- SupplementCMAPFinal.docx

## Figures and Tables

**Table 1:** Baseline characteristics for discovery and validation cohorts

Characteristic	Discovery Cohort (n = 1963)	Validation Cohort (n = 1172)
Age, mean (± SD)	66.9 (11.3)	66.0 (11.2)
Male sex, n (%)	1126 (42.6)	594 (50.7)
Race, n (%)		
White	1659 (84.0)	990 (84.5)
Black	111 (5.7)	45 (3.8)
Asian	87 (4.4)	83 (7.1)
Other/Unknown	106 (5.4)	54 (4.6)
Diagnosis, n (%)		
IPF	950 (48.4)	388 (33.1)
CTD-ILD	447 (22.8)	452 (38.6)
fHP	275 (14.0)	111 (9.5)
uILD	171 (8.7)	203 (17.3)
INSIP	120 (6.1)	18 (1.5)
Ever smoker, n (%)	1011 (51.5)	628 (53.6)
FVC %, mean (± SD)	69.5 (18.0)	78.2 (19.2)
DLCO %, mean (± SD)	46.2 (17.5)	53.9 (17.8)
Anti-fibrotic exposure, n (%)		
At blood draw, n (%)	541 (27.6)	128 (10.9)
Following blood draw, n (%)	880 (44.8)	234 (20.0)
Immunosuppressant exposure, n (%)		
At blood draw, n (%)	374 (19.1)	391 (33.4)
Following blood draw, n (%)	670 (34.1)	508 (43.3)

Abbreviations: IPF = idiopathic pulmonary fibrosis; CTD = connective tissue disease; ILD = interstitial lung disease; fHp = fibrotic hypersensitivity pneumonitis; uILD = unclassifiable interstitial lung disease; INSIP = idiopathic non-specific interstitial pneumonia; FVC = forced vital capacity; DLCO = diffusion capacity of the lung for carbon monoxide

**Table 2:** Natural indirect effects for validated proteins in discovery and validation cohorts.

Symbol	Discovery Cohort (n = 1963)				Validation Cohort (n = 1172)			
	Coefficient	95% CI	FDR P	E-value	Coefficient	95% CI Low	FDR P	E-value
AREG	−2.65	−3.45, −1.85	7.02E-09	2.00	−2.24	−3.14, −1.35	2.16E-05	1.99
CSF2	−1.12	−1.73, −0.52	1.85E-03	1.51	−1.06	−1.75, −0.37	1.22E-02	1.55
GDF15	−1.59	−2.30, 0.88	1.14E-04	1.66	−1.00	−1.75, −0.25	3.14E-02	1.53
IL5RA	−1.06	−1.72, −0.41	3.80E-03	1.49	−1.05	−1.69, −0.40	9.53E-03	1.54
ITGB6	−2.51	−3.33, −1.69	6.22E-08	1.95	−2.21	−3.16, −1.26	7.03E-05	1.98
MMP10	−1.25	−1.90, −0.59	1.08E-03	1.55	−1.17	−1.93, −0.40	1.16E-02	1.59
PLA2G10	−1.19	−1.87, −0.50	3.01E-03	1.53	−1.52	−2.27, −0.77	6.25E-04	1.72

Abbreviations: FDR = false discovery rate; CI = confidence interval

## Data Availability

Individual level data and summary statistics for this study will be made available within 6 months of publication through BioLINCC (https://biolincc.nhlbi.nih.gov/home/). Investigators interested in accessing individual-level data prior to BioLINCC release should contact Dr. Justin Oldham (oldhamj@med.umich.edu).
